# Exploring the potential relationship between short sleep risks and cognitive function from the perspective of inflammatory biomarkers and cellular pathways: Insights from population‐based and mice studies

**DOI:** 10.1111/cns.14783

**Published:** 2024-05-26

**Authors:** Yanwei You, Jinwei Li, Yang Zhang, Xingtian Li, Xinming Li, Xindong Ma

**Affiliations:** ^1^ Division of Sports Science & Physical Education Tsinghua University Beijing China; ^2^ IDG/McGovern Institute for Brain Research Tsinghua University Beijing China; ^3^ Department of Neurosurgery, West China Hospital Sichuan University Chengdu China; ^4^ Department of Vascular Surgery Fuwai Yunnan Cardiovascular Hospital, Affiliated Cardiovascular Hospital of Kunming Medical University Kunming China

**Keywords:** cellular pathways, cognitive function, inflammatory biomarkers, short sleep

## Abstract

**Aims:**

The molecular mechanism of short‐sleep conditions on cognition remains largely unknown. This research aimed to investigate associations between short sleep, inflammatory biomarkers and cognitive function in the US population (NHANES data 2011–2014) and explore cellular mechanisms in mice.

**Methods:**

Systemic immune‐inflammation index (SII) was calculated using blood‐cell based biomarkers. Further, we employed integrated bioinformatics and single‐cell transcriptomics (GSE137665) to examine how short sleep exposure influenced the molecular pathways associated with inflammation in the brain. To explore the signaling pathways and biological processes of sleep deprivation, we carried out enrichment analyses utilizing the GO and KEGG databases.

**Results:**

Population results showed that, compared with normal sleep group, severe short sleep was associated with lower cognitive ability in all the four tests. Moreover, a higher SII level was correlated with lower scores of cognitive tests. In mice study, elevated activation of the inflammatory pathway was observed in cell subgroups of neurons within the sleep deprivation and recovery sleep cohorts. Additionally, heightened expression of oxidative stress and integrated stress response pathways was noted in GABAergic neurons during sleep deprivation.

**Conclusion:**

This study contributed to the understanding of the influence of short sleep on cognitive function and its cellular mechanisms.

## Graphical abstract

Severe short sleep and higher systemic immune‐inflammation index level was associated with lower cognitive ability. Additionally, heightened expression of oxidative stress and integrated stress response pathways was noted in GABAergic neurons during sleep deprivation.

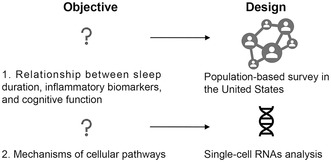

## 1 INTRODUCTION

Sleep is a fundamental physiological process essential for maintaining optimal cognitive functioning and overall health. The National Sleep Foundation (NSF) panel has annually published a recommended appropriate sleep duration (7–9 h of healthy adults), also known as the referent sleep duration.^1^ However, in recent years, the prevalence of short sleep duration has been on the rise, posing significant concerns for public health.^2^ In generally populations, short sleep duration has been robustly associated with a heightened susceptibility to accelerated aging,^3^ obesity,^4^ type 2 diabetes mellitus,^4^, ^5^ hypertension,^4^, ^6^ cardiovascular diseases,^7^ and overall mortality risk.^4^, ^8^

Furthermore, the influence of inadequate sleep duration on cognition and neuroscience represents a burgeoning area of interest,^9^, ^10^ yet remains largely unexplored. Current evidence tended to show that extreme short sleep (e.g., sleep deprivation) was associated with several neurological diseases, such as stroke,^11^ Alzheimer's,^12^ Parkinson's disease,^13^ and depression.^14^ Amyloid‐β, a protein fragment, has been identified as a key player in the relationship between short sleep and cognitive decline.^15^, ^16^ Interestingly, emerging evidence suggested that one‐night short sleep duration may be associated with alterations in the clearance or production of amyloid‐β in the brain.^17^ These findings have raised intriguing questions about the potential link between inadequate sleep and cognitive function and its underlying mechanisms.

The impact of short sleep on the body's inflammatory processes has long been recognized, with ample evidence supporting the detrimental effects of chronic sleep deprivation.^18^, ^19^ In recent years, there has been growing interest in understanding the role of inflammation in the relationship between short sleep and cognitive function.^20^, ^21^ Research indicated that inadequate sleep can trigger an inflammatory response in the body, leading to increased levels of pro‐inflammatory cytokines and other markers of inflammation.^21^, ^22^ This chronic low‐grade inflammation has been associated with cognitive decline and an increased risk of cognitive disorders such as dementia and Alzheimer's disease.^23^

Recent advances in molecular biology have shed light on the interplay between sleep duration and gene expression.^24^, ^25^ Sleep patterns have been shown to influence the expression of various genes involved in immune regulation, stress response, and neuronal function.^22^, ^26^ The application of emerging single‐cell transcriptomics presents a promising avenue for exploring and comprehending the influence of short sleep on biological systems. This innovative technique enables us to delve into the molecular intricacies of individual cells, providing unprecedented insights into how short sleep might impact various cellular functions. By deciphering the unique gene expression patterns of diverse cell types under conditions of inadequate sleep, we can gain a deeper understanding of the molecular mechanisms underlying the potential connections between short sleep and physiological processes. This approach holds the potential to uncover novel biomarkers, pathways, and regulatory networks that are affected by short sleep, shedding light on the intricate interplay between sleep duration, cellular function, and overall health.

It is worth noting that the relationship between short sleep, inflammation, and cognitive function is complex and multifactorial. Other demographic and lifestyle factors, such as age, sex, race, body mass, and physical activity, can also influence inflammation and cognitive health. Therefore, comprehensive studies that consider multiple variables are necessary to better understand the mechanisms underlying this association. Identifying the precise role of inflammation in the link between short sleep and cognitive function has important implications for sleep health. To narrow the research gap, this study seeks to contribute to the growing body of knowledge in this domain by (i) exploring the associations between short sleep risk and cognitive function from a population‐based analysis of National Health and Nutrition Examination Survey (NHANES); (ii) investigating potential changes and links to inflammatory biomarkers and cellular pathways in the mice brain caused by short sleep using the GEO data.

## 2 MATERIALS AND METHODS

### 2.1 Study design and population

The NHANES, a comprehensive and cross‐sectional nationwide health survey conducted since 1988, serves as an invaluable repository of data pertaining to the non‐institutionalized US population. The initial phase of this investigation involved interviews conducted by suitably trained professionals, who collected pertinent background information encompassing socio‐demographic profiles, medical histories, and familial lineage. Subsequently, partial participants were invited to undergo a thorough physical and medical evaluation at the Mobile Examination Center (MEC). NHANES stands out for its extensive coverage of diverse demographics, ensuring a broad representation of the population. This inclusivity enhances the robustness and generalizability of our conclusions, as the findings are based on a comprehensive cross section of individuals from various ethnic backgrounds, socioeconomic statuses, and geographic locations. As a result, the evidence derived from NHANES data is highly reliable and provides insights into the broader population's health trends and patterns. As part of the Centers for Disease Control and Prevention, the National Center for Health Statistics (NCHS) conducted the survey and the Ethics Review Board of NCHS approved the ethical endorsement.

The wealth of cognitive function measures, sleep information, and inflammatory biomarkers was available within the NHANES database for the year 2011–2014. Given the framework of our research design, based on a cross‐sectional analysis, our selected cohort comprised exclusively of adult participants, who willingly subjected themselves to the cognitive function evaluation administered during these two survey cycles. In this study, 3632 participants were initially included. After that, we expunged from our study those participants who failed to complete the requisite cognitive testing or provide adequate data regarding their sleep information. Moreover, participants missing inflammatory and covariates data were excluded. Consequently, the total sample size of 2641 cases remained for the final analysis.

### 2.2 Measurement of sleep status

NHANES included a self‐reported sleep duration questionnaire as part of its data collection process. Participants were asked to report their average number of hours of sleep obtained during a typical 24‐h period. This information was collected through interviews conducted by trained interviewers. During the survey, participants answered a question about their sleep hours routinely: “How much sleep do you get (hours) in a typical day?”^27^, ^28^ Referring to the suggestions by the National Sleep Foundation,^1^ sleep duration was categorized into normal sleep (≥7 h), mild short sleep (≥6 h, <7 h), and severe short sleep (<6 h).

### 2.3 Measurement of cognitive function

The cognitive capabilities of individuals who participated in the mobile examination component of the NHANES were assessed using a battery of neurocognitive tests specifically designed to evaluate various aspects of cognitive function. The Consortium to Establish a Registry for Alzheimer's Disease (CERAD) Word List Learning Test, the CERAD Word List Recall Test, the Animal Fluency Test, and the Digit Symbol Substitution Test (DSST) were employed for this purpose, with these tests having undergone rigorous development and validation processes within the national population.^29^, ^30^, ^31^ The utilization of these cognitive tests in the evaluation of individuals' cognitive abilities within the NHANES mobile examination component yielded comprehensive insights into their immediate and delayed learning capabilities, executive functioning, and cognitive processing speed, sustained attention, and working memory. Higher scores across these neurocognitive assessments are suggestive of enhanced cognitive performance across multiple domains. Detailed information about these four cognitive tests can be found in the Appendix S1.

### 2.4 Measurement of inflammatory biomarkers

The focus of this study revolves around the evaluation of the systemic immunity‐inflammation index (SII) as the inflammatory biomarkers. SII's advantage lies in its composite nature, incorporating multiple components such as neutrophils, lymphocytes, and platelets, providing a comprehensive assessment of inflammation. This contrasts with C‐reactive protein (CRP), which is a single‐dimensional marker. Moreover, SII demonstrates superior sensitivity and specificity compared to CRP and white blood cell (WBC) count in predicting inflammatory conditions and disease outcomes.

To effectively quantify and assess the relevant hematological parameters essential for determining the SII, the utilization of hematology analysis equipment was employed. Specifically, the Coulter DxH 800 analyzer facilitated the measurement and subsequent reporting of the lymphocyte, neutrophil, and platelet counts in units of 10^9^ cells/L. Details of the blood sample assessment can be found in the Appendix S1. The computation of the SII level hinges upon the following formula: (platelet count × neutrophils count)/lymphocytes count.^32^, ^33^, ^34^, ^35^ Referring to previous literature,^36^ SII was set as a categorical variable according to the mean value.

### 2.5 Measurement of covariates

With the aim of comprehensively examining the multifaceted relationship between sleep duration, inflammatory biomarkers, and cognitive function, our research design encompassed a deliberate selection of covariates based on prior investigations in the field.^37^, ^38^, ^39^ These chosen covariates included age, sex, ethnicity, body measurements (body mass index, BMI), and physical activity (work activity and recreational activity). Age assumed a critical role as an essential covariate, allowing for a thorough understanding of the potential influence that advancing years may exert on cognitive function and sleep patterns. Given the impact of cultural and genetic variations, we categorized ethnicity into four distinct groups—non‐Hispanic white, non‐Hispanic black, Mexican American, and other races—to capture the potential nuances and heterogeneity associated with this covariate. Furthermore, body measurements, explicitly encapsulated by BMI, served as crucial covariates in our investigation. This metric, computed as weight in kilograms divided by measured height in meters squared, provided valuable insights into the physiological dimensions that may underpin the observed associations. Physical activity, an indispensable lifestyle factor warranting consideration, was thoroughly evaluated in both work and recreational contexts. Work‐related physical activities encompass paid and unpaid employment, household chores, and yard work, capturing a wide range of occupational and domestic exertions. Additionally, recreational activity—embracing leisure time physical endeavors such as sports, fitness pursuits, and other recreational engagements—was accounted for to comprehensively assess the influence of physical activity practices on the association between sleep, SII and cognitive function. Through the Physical Activity Questionnaire in NHANES, self‐reported information about physical activity was collected. The systematic integration of covariates mentioned above might enhance the robustness and validity of study findings.

### 2.6 Statistical processing of NHANES data

In accordance with the NHANES protocol, all data were integrated into a single data set, and the masked variance was factored into the analysis along with the weighting method suggested in the protocol. Reweighting of sample weights was performed on NHANES data from 2011 to 2014, which addressed non‐response, non‐coverage, and unequal selection probabilities. We expressed continuous variables as weighted means and standard errors (SE), and categorical variables as weighted percentages. To assess relationships between sleep, SII and cognitive function, weighted linear regression analysis was employed, considering the complex survey design of NHANES. Three distinct models were constructed to provide robust statistical inference.^40^, ^41^ The crude model constituted the baseline analysis, without adjusting for any covariates. Model 1 incorporated essential demographic factors, namely age, sex, and race, to account for their potentially confounding effects on the observed associations. To expand upon Model 1, Model 2 further adjusted for additional variables, including BMI and physical activity, recognized as crucial determinants of cognitive function. Analyses were performed using R Software (version 4.2.1) and the “survey” package was used accounting for the weighting procedure.

### 2.7 Molecular and cellular pathways analysis

This study used wild‐type C57BL/6 J mice subjected to sleep restriction by extending their wakefulness into the light phase of the light–dark cycle. The brainstem, cortex, and hypothalamus were chosen as regions of interest, as they are implicated in the regulation of sleep–wake states.^42^, ^43^ To assess the impact of sleep deprivation on the cerebral microenvironment cells, we conducted single‐cell transcriptomic analyses of brain tissues (brainstem, cortex, and hypothalamus) from mice subjected to normal sleep conditions (*N* = 3), 12‐h sleep deprivation (*N* = 3), and 12‐h recovery sleep (*N* = 3). The data were sourced from the GEO database and are publicly accessible under the accession number GSE137665.^44^

Referring to previous literature,^44^ the data underwent initial normalization and log‐transformation using the NormalizeData function in Seurat. The Seurat function FindMarker was utilized to compute differential gene expression using the Wilcoxon rank‐sum test method. For data analysis, we employed quality control criteria allowing for gene count and ribosomal proportion as follows: minGene = 200, maxGene = 3000, pctMT = 20 (Figure S1A,B). We selected 2000 genes with substantial expression variation to represent the cellular transcriptional profile (Figure S1C). After dimensionality reduction and clustering, the “harmony” R package was utilized to mitigate batch effects and better capture cellular types and biological variations across different batches (Figure S1D).^45^ A total of 27 clusters were identified (Figure S1E). Clusters were visualized with uniform manifold approximation and projection (UMAP) in R package “Seurat” with default settings. Marker gene expression across different cell clusters can be found in Figure S1F. Manual cell type annotation was performed through automated literature referencing, including Astrocytes (Gfap, Aqp4, Gja1, Aldh1l1, Slc1a3, Slc1a2), Microglia (Tmem119, Cx3cr1, P2ry12, Csf1r, Fcrls), Endothelial cells (Cldn5, Flt1, Pecam1, Kdr), GABAergic neurons (Gad1, Gad2, Slc32a1, Atp1a3, Atp1b1, Camta1), Pericytes (Rgs5, Abcc9, Kcnj8), Neurons (Meg3, Rbfox3, Snap25, Stmn2, Tubb3), and Macrophages (F13a1, Ccr2, Mrc1, Cd68).^44^, ^46^

To analyze the signaling pathways and biological functions involved in post‐sleep deprivation, we conducted Gene Ontology (GO) and Kyoto Encyclopedia of Genes and Genomes (KEGG) enrichment analyses. Only results with a *p*‐adjust <0.05 were displayed. To evaluate the impact of oxidative stress, inflammation, and integrated stress response signaling pathways, we utilized the R package “Singscore” to assess the activity levels of these pathways in samples or cells.^47^

## 3 RESULTS

Table 1 presents an overview of the basic characteristic information pertaining to the participants and their cognitive function tests performance. Our study encompassed a sizable sample of 2641 individuals, with a mean age of 69.58 years. The weighted population was 48,872,568. With regard to gender distribution, 45.76% of the participants were male, while approximately 79.99% identified as non‐Hispanic white. The mean BMI of study participants was 29.14 kg/m^2^. Delving further into physical activity aspects, it was revealed that participants reported an average of 146.31 and 276.67 min of recreational activity and work activity per week, respectively. Furthermore, the mean sleep duration was 7.17 h and half (52.30%) of the participants received enough sleep (at least 7 h) recommended by the National Sleep Foundation.

**TABLE 1:** Descriptions of the demographic characteristics of NHANES (2011–2014) participants.

Continuous variables	Mean ± SE
Score of the CERAD Immediate Recall	6.55 ± 0.08
Score of the CERAD Delayed Recall	6.19 ± 0.10
Score of the Animal Fluency Test	18.15 ± 0.19
Score of the Digit Symbol Substitution Test	52.28 ± 0.58
Recreational activity (min/week)	146.31 ± 9.76
Work activity (min/week)	276.67 ± 28.05
Sleep duration (h/day)	7.17 ± 0.03
Systemic immune‐inflammation index (10^9^/L)	565.75 ± 10.64

Category variables	%
Systemic immune‐inflammation index (category)	
<544.64	60.44
≥544.64	39.56
Sleep duration (category)	
≥7 (Normal sleep)	52.30
≥6 (Mild short‐sleep)	32.44
<6 (Severe short‐sleep)	15.25
Age	
<65	32.23
[65, 72)	35.61
≥72	32.16
Sex	
Male	45.76
Female	54.24
Race/ethnicity	
Non‐Hispanic White	79.99
Non‐Hispanic Black	8.11
Mexican American	3.42
Other Race/ethnicity	8.48
Body mass index (kg/m^2^)	
<25	26.03
[25, 30)	36.16
≥30	37.81

*Note*: Weighted Mean ± SE for continuous variables and weighted percentage for category variables.

Table 2 demonstrates the relationship between short sleep risks and different cognitive function tests. As for the crude model, short sleep was associated with lower test scores. Taken normal sleep group as the reference, mild short‐sleep and severe short‐sleep were both negatively associated with the score of the CERAD Immediate Recall Test [*β* (95% CI): −0.211 (−0.400, −0.022), *p* = 0.030; *β* (95% CI): −0.515 (−0.782, −0.247), *p* < 0.001], score of the CERAD Delayed Recall Test [*β* (95% CI): −0.039 (−0.356, 0.278), *p* = 0.804; *β* (95% CI): −0.448 (−0.852, −0.045), *p* = 0.031], score of the Animal Fluency Test [*β* (95% CI): −1.226 (−2.138, −0.314), *p* = 0.010; *β* (95% CI): −2.115 (−3.233, −0.997), *p* < 0.001], score of the Digit Symbol Substitution Test [*β* (95% CI): −2.983 (−5.301, −0.664), *p* = 0.013; *β* (95% CI): −6.559 (−9.840, −3.278), *p* < 0.001]. After adjusting for age, sex, race, Model 1 showed similar associations as the crude model. As for the fully adjusted model (Model 2), it was also identified that severe short‐sleep was negatively correlated with cognitive function [for CERAD Immediate Recall Test, *β* (95% CI): −0.409 (−0.664, −0.154), *p* = 0.003; for CERAD Delayed Recall Test, *β* (95% CI): −0.303 (−0.643, 0.037), *p* = 0.078; for Animal Fluency Test, *β* (95% CI): −1.135 (−2.302, 0.032), *p* = 0.056; for Digit Symbol Substitution Test, *β* (95% CI): −3.466 (−6.407, −0.525), *p* = 0.023].

**TABLE 2:** Associations between short‐sleep condition and cognitive function tests.

	Crude model^a^		Model 1^b^		Model 2^c^	
	*β* (95% CI)	*p*‐value	*β* (95% CI)	*p*‐value	*β* (95% CI)	*p*‐value
Score of the CERAD Immediate Recall Test						
Normal sleep	Reference		Reference		Reference	
Mild short‐sleep	−0.211 (−0.400, −0.022)	0.030	−0.203 (−0.405, −0.001)	0.049	−0.196 (−0.395, 0.002)	0.052
Severe short‐sleep	−0.515 (−0.782, −0.247)	<0.001	−0.406 (−0.660, −0.152)	0.003	−0.409 (−0.664, −0.154)	0.003
Score of the CERAD Delayed Recall Test						
Normal sleep	Reference		Reference		Reference	
Mild short‐sleep	−0.039 (−0.356, 0.278)	0.804	−0.043 (−0.366, 0.280)	0.787	−0.037 (−0.361, 0.287)	0.815
Severe short‐sleep	−0.448 (−0.852, −0.045)	0.031	−0.297 (−0.651, 0.057)	0.097	−0.303 (−0.643, 0.037)	0.078
Score of the Animal Fluency Test						
Normal sleep	Reference		Reference		Reference	
Mild short‐sleep	−1.226 (−2.138, −0.314)	0.010	−0.953 (−1.749, −0.157)	0.021	−0.828 (−1.638, −0.018)	0.045
Severe short‐sleep	−2.115 (−3.233, −0.997)	<0.001	−1.279 (−2.420, −0.137)	0.030	−1.135 (−2.302, 0.032)	0.056
Score of the Digit Symbol Substitution Test						
Normal sleep	Reference		Reference		Reference	
Mild short‐sleep	−2.983 (−5.301, −0.664)	0.013	−1.978 (−4.133, 0.177)	0.070	−1.602 (−3.551, 0.346)	0.102
Severe short‐sleep	−6.559 (−9.840, −3.278)	<0.001	−3.771 (−6.736, −0.806)	0.015	−3.466 (−6.407, −0.525)	0.023

Abbreviation: CI, confidence interval.

^a^ Crude model, no covariates were adjusted.

^b^ Model 1, age, sex, race were adjusted.

^c^ Model 2, age, sex, race, body mass index, work activity, and recreational activity were adjusted.

Furthermore, in Table 3, the association between inflammation indicated by systemic immunity‐inflammation index (SII) and cognitive function was examined. In the crude model, higher SII level was found to be significantly associated with lower scores of CERAD Immediate Recall [*β* (95% CI): −0.200 (−0.397, −0.003), *p* = 0.047] and CERAD Delayed Recall Test [*β* (95% CI): −0.463 (−0.783, −0.143), *p* = 0.006]. After adjusted for covariates in the Model 1, SII was identified to be negatively associated with scores of the CERAD Delayed Recall [*β* (95% CI): −0.376 (−0.671, −0.081), *p* = 0.014] and Digit Symbol Substitution Test [*β* (95% CI): −1.934 (−3.784, −0.083), *p* = 0.041]. In the Model 2, there was also a negative correlation between SII and score of the CERAD Delayed Recall Test [*β* (95% CI): −0.375 (−0.678, −0.071), *p* = 0.018].

**TABLE 3:** Associations between systemic immune‐inflammation index and cognitive function tests.

	Crude model^a^		Model 1^b^		Model 2^c^	
	*β* (95% CI)	*p*‐value	*β* (95% CI)	*p*‐value	*β* (95% CI)	*p*‐value
Score of the CERAD Immediate Recall Test						
<544.64	Reference		Reference		Reference	
≥544.64	−0.200 (−0.397, −0.003)	0.047	−0.148 (−0.330, 0.034)	0.106	−0.144 (−0.332, 0.044)	0.127
Score of the CERAD Delayed Recall Test						
<544.64	Reference		Reference		Reference	
≥544.64	−0.463 (−0.783, −0.143)	0.006	−0.376 (−0.671, −0.081)	0.014	−0.375 (−0.678, −0.071)	0.018
Score of the Animal Fluency Test						
<544.64	Reference		Reference		Reference	
≥544.64	−0.489 (−1.265, 0.286)	0.207	−0.580 (−1.255, 0.095)	0.089	−0.534 (−1.219, 0.150)	0.120
Score of the Digit Symbol Substitution Test						
<544.64	Reference		Reference		Reference	
≥544.64	−1.819 (−4.062, 0.424)	0.108	−1.934 (−3.784, −0.083)	0.041	−1.745 (−3.702, 0.211)	0.078

Abbreviation: CI, confidence interval.

^a^ Crude model, no covariates were adjusted.

^b^ Model 1, age, sex, race were adjusted.

^c^ Model 2, age, sex, race, body mass index, work activity, and recreational activity were adjusted.

**FIGURE 1:**
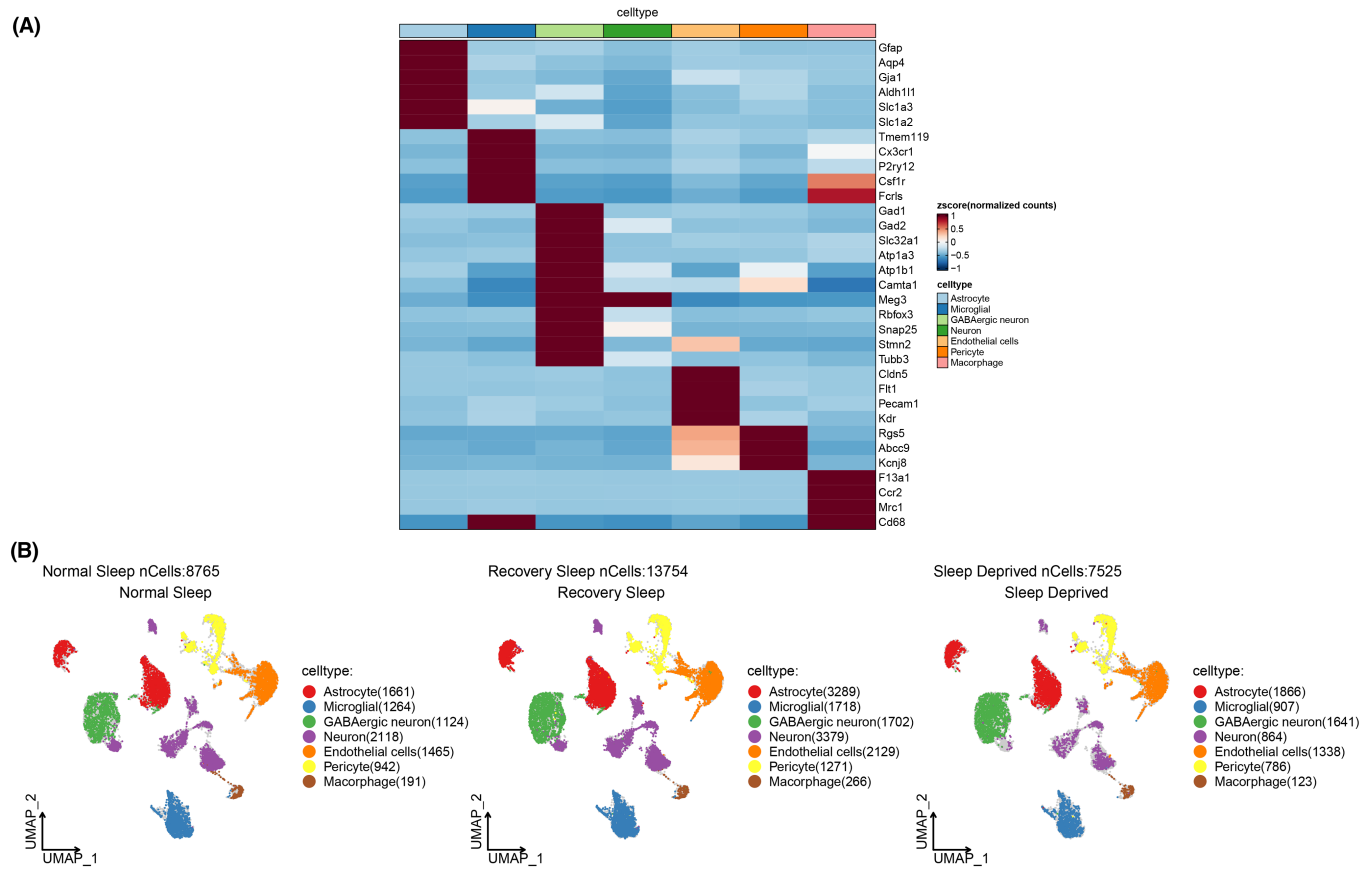
Visualization of cell subgroups' distribution using marker gene expression for normal sleep, 12‐h sleep deprivation, and 12‐h recovery sleep conditions. (A) Expression of marker genes visualized; (B) UMAP visualization of cell subgroup clustering.

We investigated the impact of sleep deprivation on cellular microenvironment in the brain through single‐cell transcriptomics using the data from the GEO database. We presented the cell subgroups after clustering analysis, which encompassed Astrocytes, Microglia, GABAergic neurons, Neurons, Endothelial cells, Pericytes, and Macrophages (Figure 1A). As illustrated in Figure 1B, UMAP clustering displayed the cellular distribution under normal sleep conditions (cell count = 8765), 12‐h sleep deprivation (cell count = 13,754), and after 12‐h recovery sleep (cell count = 7525).

**FIGURE 2:**
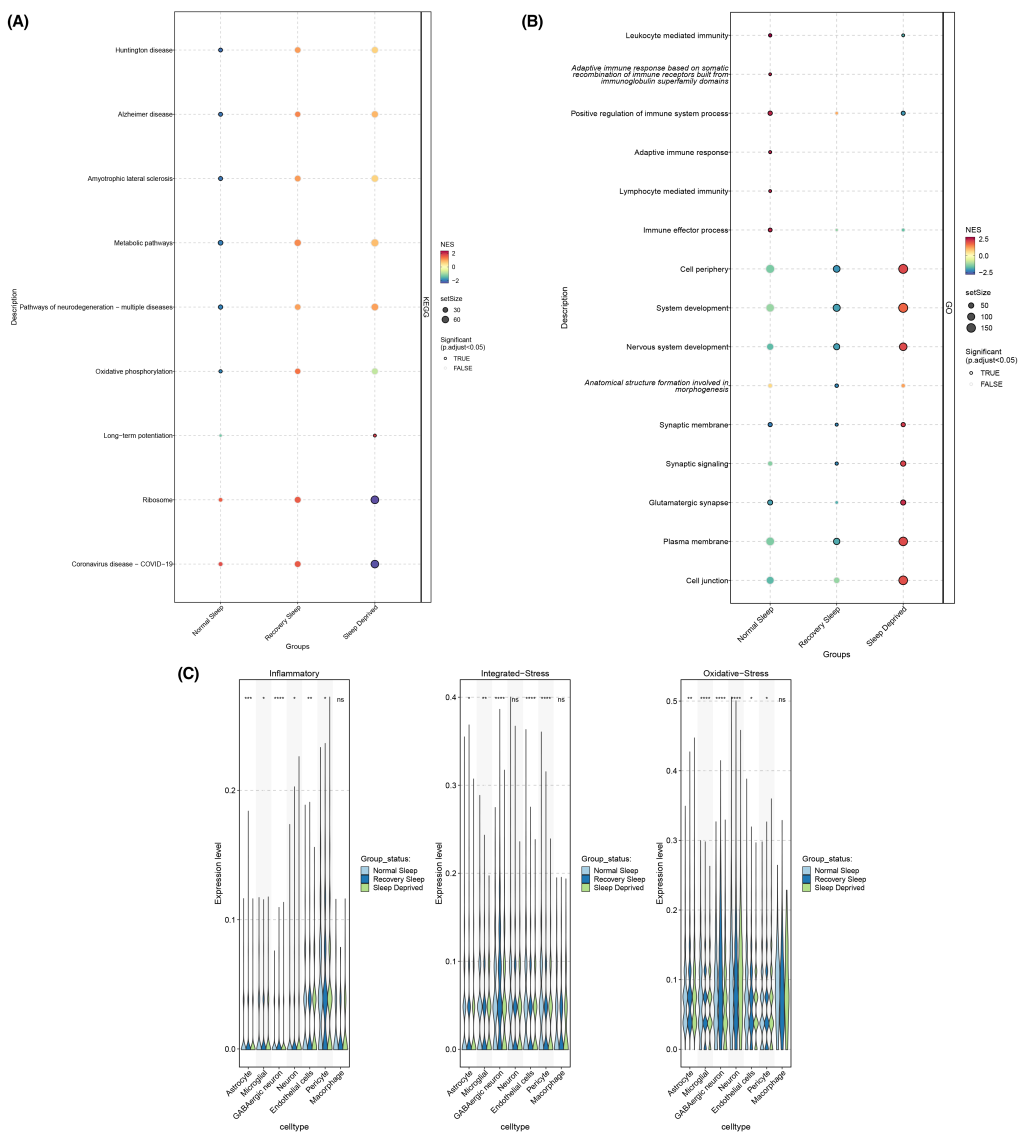
Enrichment analysis of biological functions for normal sleep, 12‐h sleep deprivation, and 12‐h recovery sleep. (A) Pathway enrichment analysis; (B) Enriched biological processes; (C) Scoring of gene sets related to oxidative stress, inflammation, and integrated stress response.

**FIGURE 3:**
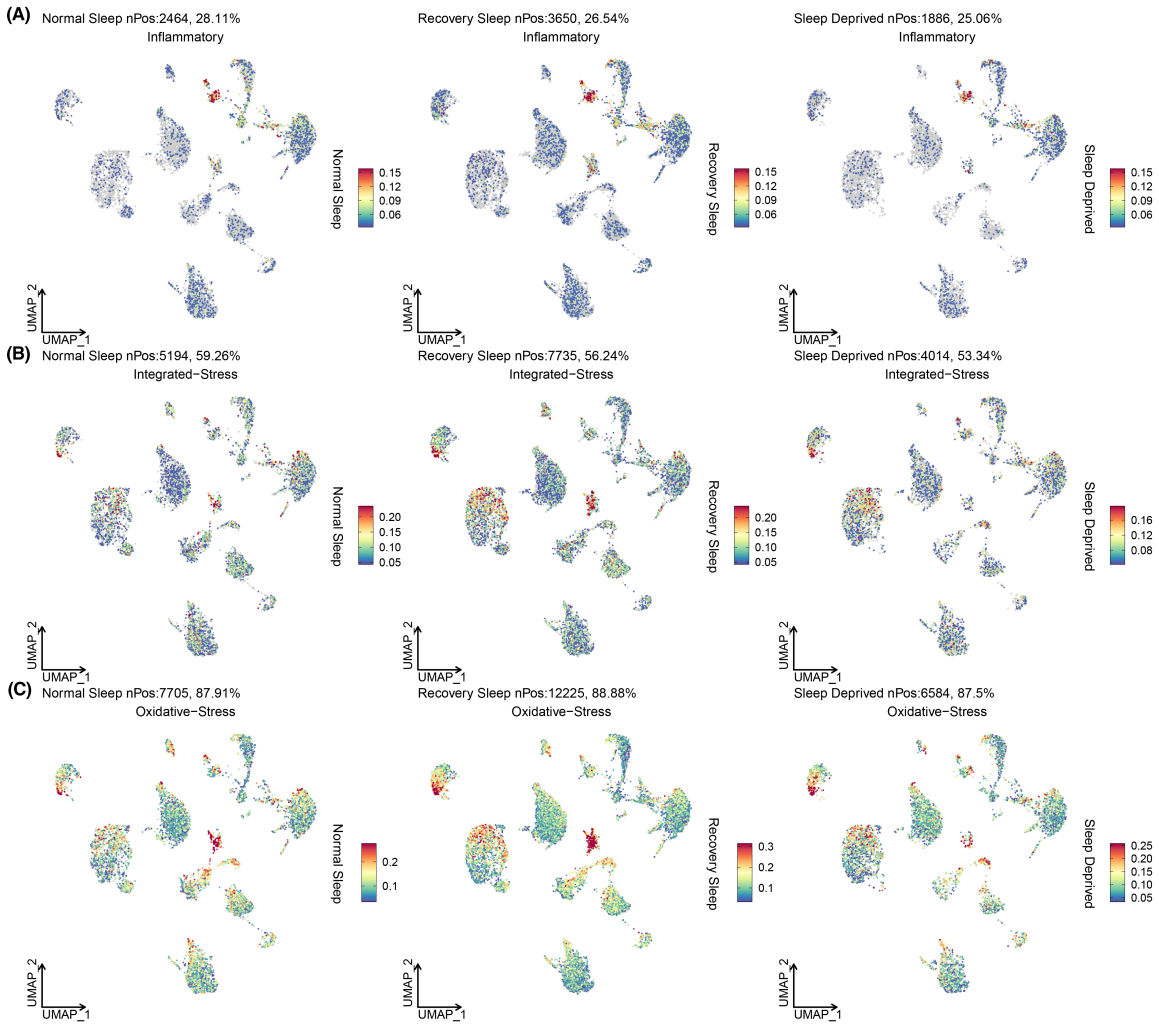
UMAP visualization of the distribution of oxidative stress, inflammation, and integrated stress response gene sets across cell subgroups for normal sleep, 12‐h sleep deprivation, and 12‐h recovery sleep. (A) Visualization of oxidative stress; (B) Visualization of inflammation; (C) Visualization of integrated stress response.

Subsequently, we conducted pathway enrichment analysis. Compared to the normal sleep group, the sleep deprivation and recovery sleep groups were predominantly enriched in pathways such as Huntington's disease, Alzheimer's disease, Metabolic pathways, and Pathways of neurodegeneration—multiple diseases (Figure 2A). Additionally, we observed that biological functions in the sleep deprivation and recovery sleep groups were significantly enriched in categories like Cell periphery, System development, Nervous system development, Anatomical structure formation involved in morphogenesis, Synaptic membrane, Synaptic signaling, and Glutamatergic synapse (Figure 2B). Moreover, using scoring based on gene sets related to oxidative stress, inflammation, and integrated stress response, we identified differential expression across various cell subgroups (Figure 2C). Details of gene sets of the above three pathways are shown in Table S1. Finally, we visualized the distribution of these three signaling pathways on the UMAP. Inflammatory pathway activation was found to be elevated in Neurons of the sleep deprivation and recovery sleep groups (Figure 3A). Oxidative stress and integrated stress response pathways exhibited heightened expression in GABAergic neurons under sleep deprivation (Figure 3B,C).

## 4 DISCUSSION

This study investigated the association between short sleep duration and cognitive function while exploring potential links to inflammatory biomarkers and cellular pathways. From a nationwide population, we found that severe short sleep (less than 6 h) was negatively associated with cognitive function. Moreover, the role of systemic inflammation was identified, which showed that SII calculated by blood‐cell biomarkers was also negatively associated with cognitive test scores. Furthermore, we conducted an analysis of single‐cell transcriptomics in mice's brain, and detected that inflammatory, oxidative stress and integrated stress response pathways were activated in the sleep deprivation group.

The interplay between sleep and cognition has attracted significant attention due to its potential implications for overall health. Sleep deprivation can trigger an inflammatory cascade within the brain.^48^ This is evidenced by the activation of microglia, the brain's immune cells, which initiate the release of pro‐inflammatory cytokines and other molecules.^49^, ^50^ These inflammatory molecules can have detrimental effects on neuronal health and cognitive function. The increased inflammatory response disrupts synaptic plasticity, impairs neurotransmitter balance, and affects neuronal communication, ultimately contributing to cognitive deficits. Moreover, inflammation may also affect the blood–brain barrier, making it more permeable and allowing inflammatory molecules to enter the brain.^51^ This can further exacerbate neuroinflammation and neurodegenerative processes, potentially accelerating cognitive decline.^52^ Additionally, chronic inflammation resulting from prolonged sleep deprivation can lead to oxidative stress and cellular damage,^53^, ^54^ further exacerbating cognitive decline.

The negative correlation between severe short sleep and cognition in people was further verified in mice. This study showed that pathways such as Huntington's disease, Alzheimer's disease, metabolic pathways, and pathways of neurodegeneration—multiple diseases were enriched in the sleep deprivation and recovery sleep mice group. Considering that sleep deprivation leads to a pro‐inflammatory shift, with elevated levels of pro‐inflammatory cytokines and a blunted anti‐inflammatory response.^49^, ^55^ This imbalance not only compromises the immune system's ability to combat infections but also contributes to the development of chronic inflammatory conditions, ranging from metabolic disorders to neurodegenerative diseases.^56^, ^57^

Our study illuminated activations of fundamental cellular pathways, including oxidative stress and integrated stress response pathways, in GABAergic neurons due to sleep deprivation. GABAergic neurons play pivotal roles in regulating sleep, storing memory, and safeguarding the body against stressors.^58^ The impact of sleep deprivation extends to the realm of oxidative stress pathways, which regulate the equilibrium between cellular oxidative damage and antioxidant defenses. Sleep plays a crucial role in oxidative stress management by enhancing antioxidant enzyme activity and reducing reactive oxygen species (ROS) production.^59^, ^60^ Sleep deprivation disturbs this equilibrium, leading to an overabundance of ROS and inadequate antioxidant defense.^61^, ^62^ This oxidative imbalance culminates in cellular damage, impacting essential biomolecules and contributing to the onset and progression of neurological disorders.^63^

The interconnection between sleep deprivation and integrated stress response (ISR) pathways is equally profound. ISR refers to oxidative stress, amino acid deficiency, endoplasmic reticulum stress, and unfolded protein accumulation. Key kinases such as GCN2, PKR, PERK, and HRI induce cellular adaptive responses mediated by eukaryotic initiation factor 2 (eIF2α) phosphorylation and transcription factor 4 (ATF4) activation.^64^ Studies have shown that integrated stress response is closely related to a variety of diseases, including obesity, arthritis, tumors, and neurodegenerative diseases.^65^, ^66^ But its role in sleep has been unclear. It can be assumed that sleep is integral to the proper functioning of these pathways, as it enables cellular repair and the replenishment of resources necessary for stress adaptation. Sleep deprivation disrupts this balance, leading to dysregulated stress hormone levels, altered stress response cascades, and compromised cellular resilience. Consequently, cellular health becomes compromised, increasing vulnerability to stress‐related diseases and diminishing the body's capacity to cope with physiological challenges.

This study boasts several notable strengths that have contributed to its scientific robustness. Leveraging data sourced from the NHANES, a comprehensive initiative overseen by the Centers for Disease Control and Prevention, we evaluated the health dynamics of the broader noninstitutionalized population in the United States. The implementation of stratified, multistage probability cluster sampling within the NHANES framework strengthened the generalizability of our findings to embrace a diverse cross‐section of the population. The population‐based study sought to identify the relationship between short sleep risk and cognitive function from a behavioral perspective. To enrich the depth of our insights, subsequent animal studies aimed to elucidate the molecular effects of sleep deprivation on brain function, bridging insights from human cohorts with mechanistic details. Unlike conventional bulk RNA quantification, our employment of single‐cell transcriptomics analysis provided a nuanced perspective by cell‐specific differentials and illuminating pathways that undergo modulation due to sleep deprivation. This study specifically identified the role of oxidative stress and ISR pathways activated in GABAergic neurons caused by sleep deprivation.

However, it is essential to acknowledge the limitations. First, given the cross‐sectional nature of NHANES, establishing causal relationships between abbreviated sleep duration and cognitive capacity remains challenging within this study design. Secondly, the study's scope is narrowed by the NHANES protocol, which mostly included participants of non‐Hispanic White ethnicity and did not consider the influence of medication use, psychiatric conditions, and chronic diseases. Thirdly, self‐reported sleep duration may introduce subjective bias into our study. Objective assessment methods, such as accelerometers and polysomnography (can both assess sleep duration and quality), may enhance the accuracy of sleep status evaluation. Fourthly, the mouse samples used for sequencing are too small. More experimental animal numbers and RNAscope in situ hybridization assays are helpful to further confirm the conclusions of this study. Finally, while one of the major strengths of this study is the integration of human population data with animal models, tightening the connection between the results of both could enhance persuasiveness.

## 5 CONCLUSION

At the core of these findings is the interplay between short sleep, cognitive function, and inflammation pathways. This study first identified the association between risks of short sleep and cognitive function from a nationwide cohort, and then detected that blood‐cell based systemic inflammation was involved in this relationship. Moreover, mice study further explained the mechanisms underlying these observations, indicating that activation of inflammatory, oxidative stress, and integrated stress response pathways were involved in the sleep loss process. Further research is warranted to validate our findings in human populations or provide additional explanations to enhance the reliability and reproducibility of our study results. Further human studies can use serum transcriptome data to identify biomarkers influencing the activation of inflammatory pathways. Animal studies can use behavioral tests such as water mazes or new object recognition tests to observe cognitive changes caused by sleep deprivation in mice, which would contribute to establishing causal relationships between the human population and animal models.

## AUTHOR CONTRIBUTIONS

Yanwei You: Conceptualization, Conducting Experiments, Formal Analysis, Writing‐Original Draft Preparation, Writing—Review & Editing. Jinwei Li: Conceptualization, Conducting Experiments, Formal Analysis, Writing—Review & Editing. Yang Zhang: Conceptualization, Conducting Experiments, Formal Analysis, Writing—Review & Editing. Xingtian Li: Formal Analysis, Literature Searches. Xinming Li: Formal Analysis, Writing‐Review & Editing. Xindong Ma: Conceptualization, Funding Acquisition, Supervision. All authors have read and agreed to the published version of the manuscript.

## CONFLICT OF INTEREST STATEMENT

The authors declare no conflicts of interest.

## Supporting information


Appendix S1

## Data Availability

The data that support the findings of this study are available from the corresponding author upon reasonable request.
