# Supplementary material for: Exploring the potential relationship between short sleep risks and cognitive function from the perspective of inflammatory biomarkers and cellular pathways: Insights from population‐based and mice studies
Source: CNS Neurosci Ther. 2024 May 26;30(5):e14783. doi: 10.1111/cns.14783 (PMC11128714; doi:10.1111/cns.14783)
Supplement: Supplementary file 1 — Appendix S1 [file CNS-30-e14783-s001.docx]

**Details about the cognitive ability tests**

To begin with, the CERAD Word List Learning and Recall tests are particularly aimed at assessing an individual's immediate and delayed learning abilities when presented with new verbal information. These tests consist of three consecutive learning trials and a subsequent delayed recall assessment. The scores for the delayed recall range from 0 to 10, whereas the total score across all three trials ranges from 0 to 30. By employing these tests, researchers sought to gauge participants' aptitude for acquiring and retaining verbal knowledge over time.

Furthermore, the Animal Fluency test was incorporated as a component targeting executive function. This particular evaluation measures an individual's ability to generate categorical verbal responses within a specific timeframe. The resulting score for the Animal Fluency test falls within the range of 3 to 39. Through this assessment, researchers aimed to gain insights into participants' proficiency in deploying cognitive flexibility and strategic thinking when producing words related to a specified semantic category.

The Digit Symbol Substitution Test (DSST), derived from the Wechsler Adult Intelligence Scale (WAIS-III), comprises a performance module designed to evaluate several cognitive domains simultaneously. Specifically, the DSST provides valuable metrics pertaining to processing speed, sustained attention, and working memory. Scores on this test span the range of 0 to 105. Higher scores obtained in all the aforementioned assessments are indicative of superior cognitive functioning and performance across various cognitive domains.

In summary, the utilization of the CERAD Word List Learning Test, the CERAD Word List Recall Test, the Animal Fluency test, and the DSST in the evaluation of individuals' cognitive abilities within the NHANES mobile examination component yielded comprehensive insights into their immediate and delayed learning capabilities, executive functioning, and cognitive processing speed, sustained attention, and working memory. Higher scores across these neurocognitive assessments are suggestive of enhanced cognitive performance across multiple domains.

**Details about the blood sample assessment**

The establishment and management of the biospecimen collection process within the confines of the mobile examination center (MEC) played a pivotal role in ensuring the efficacy and consistency of laboratory measurements across various survey locations. This encompassed the gathering, processing, storage, and transportation of diverse types of specimens, including blood and urine, among others. By virtue of its controlled environment, the MEC served as an ideal setting where laboratory analyses could be carried out under standardized conditions at each survey location. In order to facilitate specific laboratory procedures, participants were required to observe a fasting period of 9 hours prior to their respective examination sessions. The enforcement and verification of this fasting status were supervised by proficient MEC phlebotomists just before the commencement of the blood collection process. To delve into the participants' hematological profiles, a comprehensive evaluation known as the complete blood count was executed. This analysis involved the utilization of EDTA blood tubes supplied by participants and subjected to scrutiny using the Coulter® DxH 800 analyzer. Through this instrument, various parameters pertaining to blood composition and cellular elements were assessed with precision and accuracy. More laboratory procedures can be found at https://wwwn.cdc.gov/nchs/data/nhanes/2013-2014/manuals/2013_MEC_Laboratory_Procedures_Manual.pdf.


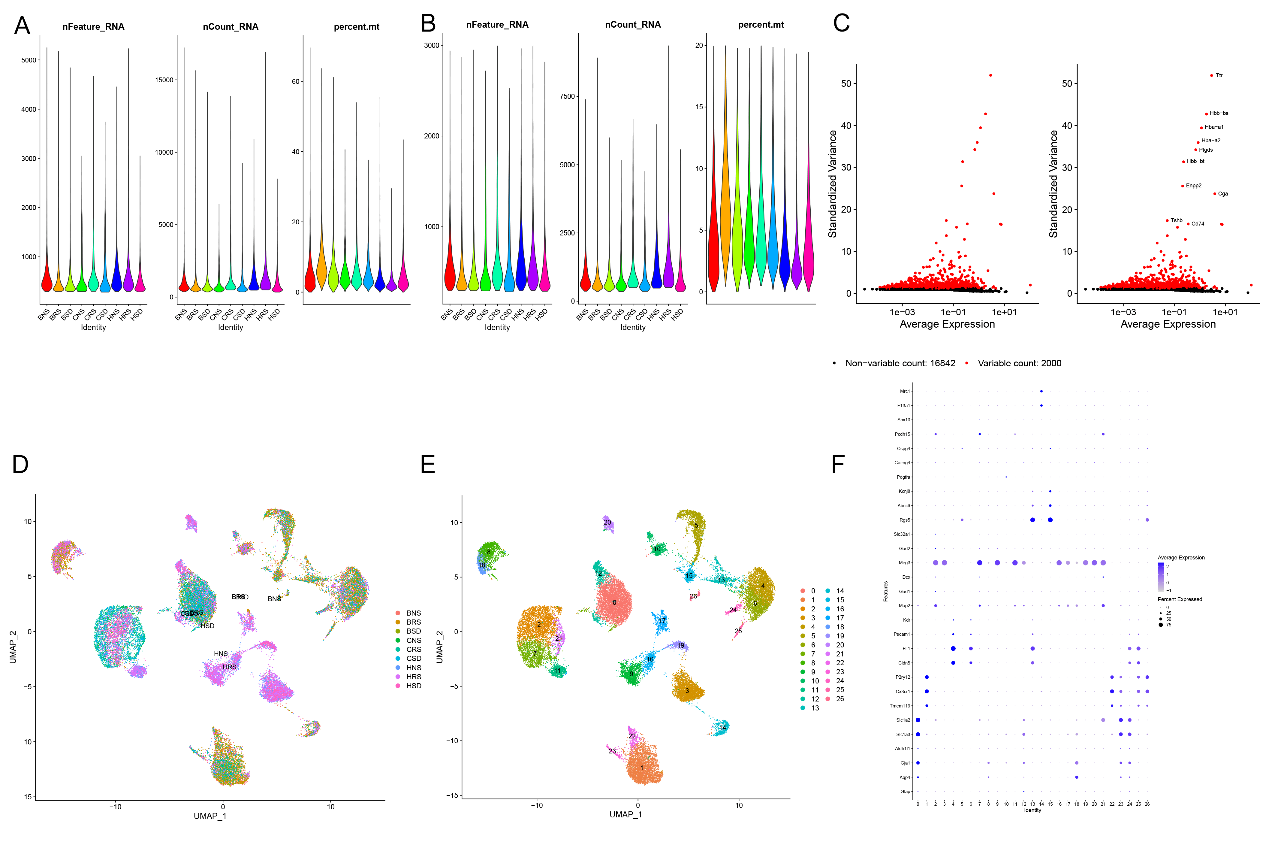
Supplementary Figure 1. Single-cell quality control processing pipeline. A. Filtering criteria for gene count and ribosomal ratio before; B. Filtering criteria for gene count and ribosomal ratio after; C. Selection of highly variable genes; D. Distribution of samples after batch correction; E. Clusters of cells after clustering; F. Marker gene expression across different cell clusters.

Supplementary Table 1. Details of gene sets of three pathways: Inflammatory, oxidative stress and integrated stress response pathways.

| WP_Inflammatory_pathways | WP_oxidative_stress_pathways | GOBP_integrated_stress_response_pathways |
| --- | --- | --- |
| Cd28 | Cat | Abca7 |
| Cd40 | Cyba | Agr2 |
| Cd40lg | Cyp1a1 | Atf4 |
| Cd80 | Fos | Bok |
| Cd86 | Gabpb1 | Dele1 |
| Col1a1 | Gclc | Eif2ak1 |
| Col1a2 | Gpx1 | Eif2ak3 |
| Col3a1 | Gpx3 | Eif2ak4 |
| Fn1 | Gsr | Eif2s1 |
| Ifng | Gstt2 | Impact |
| Il2 | Hmox1 | Map3k20 |
| Il2ra | Junb | Nck1 |
| Il2rb | Maoa | Nck2 |
| Il2rg | Mapk10 | Nfe2l2 |
| Il4 | Mapk14 | Oma1 |
| Il4ra | Mgst1 | Ptpn1 |
| Il5 | Mt1 | Ptpn2 |
| Il5ra | Nfix | Qrich1 |
| Lama5 | Nfkb1 | Tmed2 |
| Lamb1 | Nqo1 | Tmem33 |
| Lamb2 | Sod1 |  |
| Lamc1 | Sod2 |  |
| Lamc2 | Sod3 |  |
| Lck | Sp1 |  |
| Thbs1 | Txn2 |  |
| Thbs3 | Txnrd1 |  |
| Tnfrsf1a | Txnrd2 |  |
| Tnfrsf1b | Ugt1a1 |  |
| Vtn |  |  |
| Zap70 |  |  |
